# Comparison of the Change in Diastolic Dysfunction after Transcatheter Atrial Septal Defect Closure between Asymptomatic Younger and Older Age Groups

**DOI:** 10.3390/jcm9113637

**Published:** 2020-11-12

**Authors:** Lucy Youngmin Eun, Han Ki Park, Jae Young Choi

**Affiliations:** 1 Division of Pediatric Cardiology, Department of Pediatrics, Yonsei University College of Medicine, Seoul 03722, Korea; lucyeun@yuhs.ac; 2 Division of Cardiovascular Surgery, Department of Thoracic and Cardiovascular Surgery, Yonsei University College of Medicine, Seoul 03722, Korea

**Keywords:** atrial septal defect, adult, transcatheter device closure, echocardiography, LA, LV

## Abstract

Transcatheter device closure of a secundum atrial septal defect (ASD) is followed by a noticeable change in the left atrium (LA) and left ventricle (LV) over a long-term follow-up. This study aimed to assess the remodeling of the atrial and ventricular myocardium with respect to diastolic function in adult ASD patients. Around 38 asymptomatic patients (age: 48.6 ± 17.1 years, range: 23–69 years) diagnosed with ASD during routine health check-up and who underwent ASD device closure with the Amplatzer septal occluder were included in this retrospective study, and their medical records (containing echocardiographic data) were reviewed. The defect size was 21.77 + 6.79 mm, while the balloon measurement of the defect was 24.29 ± 6.64 mm. The patients were divided into two groups, namely, Group I (with 18 patients aged <50 years [mean: 33.06 ± 9.43 years] and Group II (with 20 patients aged ≥50 years [mean: 62.55 ± 7.54 years]). Comparison of echocardiographic data collected before closure and at a follow-up 2 years later revealed significant differences between pre-closure and post-closure states in the left ventricular end-diastolic dimension (40.76 ± 3.28 vs. 43.39 ± 3.52), left ventricular mass (99.64 ± 28.81 vs. 116.57 ± 32.03), and right ventricular pressure (36.88 ± 12.20 vs. 31.81 ± 11.11). Tissue Doppler measurements were significantly decreased 2 years after closure, while the post-closure E/E’ was higher than the pre-closure E/E’ (11.58 ± 4.80 vs. 8.79 + 3.19, *p* < 0.005). In both groups, mitral A and tissue Doppler E’, A’, and S’ values decreased post-closure, while the E/E’ was higher in Group II than in Group I at both pre-closure and long-term follow-up measurements (pre-closure: 9.60 ± 5.15 vs. 7.41 ± 1.42, *p* < 0.003; post-closure: 13.03 + 4.05 vs. 10.49 ± 3.95, *p* < 0.02). The LA size and LV function exhibited recognizable remodeling after transcatheter ASD closure. Because the LV preload elevation (i.e., E/E’) after ASD closure can be pronounced in older patients, caution should be provided more in older patients than in younger patients. Hence, it may be beneficial to close the ASD at an earlier age in adults even after a late diagnosis; a relatively younger age may be more suited for adaptation to the remodeling process to protect the myocardial function. Careful consideration should be given to the previous underloaded left heart after long-term closure.

## 1. Introduction

In recent years, an increasing number of cases with atrial septal defect (ASD) have been treated using transcatheter device occlusion. Beneficial for hemodynamic improvement, it is a safe procedure that is associated with lesser complications than conventional surgical procedures [1,2]. However, some elderly patients develop diastolic heart failure (HF) after occlusion [3,4]. 

Left-to-right inter-atrial shunting through the ASD causes right and left heart volume overload [5], and changes in the left atrium (LA) and left ventricle (LV) are observed during a long-term follow-up after transcatheter closure of ASD. Moreover, owing to age-related differences in LV diastolic dysfunction, the baseline hemodynamics and hemodynamic changes induced by transcatheter ASD closure are different between younger and older patients [6]. Therefore, it is necessary to clarify whether HF in elderly transcatheter occlusion patients is related to senile changes or to the impact of device closure. 

The purpose of this study was to assess cardiac function including diastolic function after transcatheter atrial septal defect closure in asymptomatic younger and older patients with ASD.

## 2. Experimental Section

### Patient Enrollment and Data Measurement

Thirty-eight patients (age: 48.6 ± 17.1 years, range: 23–69 years) incidentally diagnosed with ASD during routine health check-up, and who were indicated for ASD treatment and underwent ASD closure with the Amplatzer Septal Occluder, were included in this retrospective study. All the patients were asymptomatic. 

The patients’ medical records, including the echocardiographic data collected before closure and 2 years after closure during a follow-up, were reviewed. The patients were divided into two groups according to their ages: Group I consisted of 18 patients <50 years old (33.06 ± 9.43 years) and Group II consisted of 20 patients ≥50 years old (62.55 ± 7.54 years). 

The echocardiographic data comprised the LV inflow velocity, early and late mitral inflow velocities with the ratio of early diastole to atrial contraction (E/A), tissue Doppler image (TDI) findings, and all conventional measurements such as the LV and right ventricular (RV) diameters, LV ejection fraction (LVEF), LV mass, and LA volume. The LVEF was calculated using the Teichholz formula, while the mitral inflow pattern was assessed using the peak velocity during E and A, the ratio of E to A, and the E deceleration time. Myocardial velocities were obtained by tissue Doppler measurements (TDI) of the septal E’, septal A’, septal S’, septal E/E’, lateral E’, lateral A’, lateral S,’ and lateral E/E’, then all the TDI values were presented with the average of septal and lateral measurements.

The pre- and post-closure data were compared. Furthermore, these data were also compared between the two groups. 

## 3. Study Design and Statistical Analysis

All statistical analyses were performed using SPSS version 23.0 for Windows (SPSS Inc., Chicago, IL, USA). Data are expressed as means ± SD. Paired *t* tests were used to analyze changes in the continuous variables between baseline and follow-up assessments; accordingly, the pre-closure and post-closure baseline characteristics were compared using a paired *t* test. Differences between Groups I and II were assessed using an unpaired *t* test. The Wilcoxon signed-rank test was used to compare variables between the baseline and follow-up assessments. *p*-values <0.05 were considered statistically significant.

## 4. Ethics Statement

This study was approved by the Yonsei University College of Medicine Institutional Review Board and the Research Ethics Committee of Severance Hospital (study approval number: 2020-0699-001). All research was performed in accordance with the relevant guidelines and regulations. The requirement for written informed consent was waived by the Institutional Review Board owing to the retrospective study design.

## 5. Results

Thirty-eight patients were indicated for ASD closure. The defect size was 21.77 + 6.79 mm and the balloon measurement was 24.29 ± 6.64 mm, with RV volume overload and without pulmonary hypertension. 

The post-closure measurements of left ventricular end-diastolic dimension (LVEDD) and the LV mass were greater than their pre-closure measurements. The post-closure RV pressure was lower than the pre-closure RV pressure (Table 1). LA volume and the LA volume index increased after closure, although, this increase was not statistically significant. However, the older age patients showed different trend of LA volume and LA volume index over time rather than younger age patients (Table 2). The myocardial velocities significantly decreased after closure. Furthermore, the post-closure measurements of E’, A’, and S’ were lower than their pre-closure measurements. The estimated post-closure LV filling pressure (E/E’) was higher than the pressure in the pre-closure state (Table 1). 

**Figure 1:**
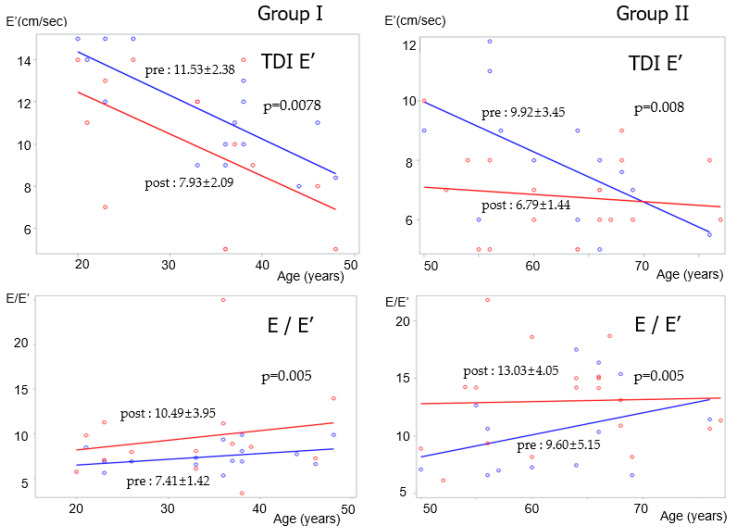
TDI E’ and E/E’ data as a function of age before and after atrial septal defect closure in Group I (N = 18) and Group II (N = 20). TDI: tissue Doppler imaging.

In Group I, the post-closure measurements of the LVEDD, LV mass, and mass index were higher than their pre-closure measurements. The post-closure RV pressure was lower than the pre-closure RV pressure. The post-closure measurements of TDI E’ and S’ were lower than their pre-closure measurements; however, A’ was higher at post-closure than at pre-closure. The estimated post-closure E/E’ was higher than the pre-closure E/E’ (Table 2) (Figure 1).

In Group II, the pre-closure measurements of LVEDD, LV mass, and mass index were higher than their pre-closure measurements. The post-closure RV pressure was lower than the pre-closure RV pressure. The TDI E’ and S’ at post-closure were lower than those at pre-closure; however, the post-closure A’ was higher than the pre-closure A’. The estimated post-closure E/E’ was higher than the pre-closure E/E’ (Table 2) (Figure 1).

With conventional diastolic velocities, significant differences were observed in the inflow velocity of late atrial kick with mitral A between Groups I and II at both pre- and post-closure. Regarding tissue Doppler myocardial velocities, E’ decreased at post-closure in Groups I (pre-closure: 11.53 ± 2.38 vs. post-closure: 7.93 ± 2.09, *p* = 0.008) (Figure 1) and II (pre-closure: 9.92 ± 3.45 vs. post-closure: 6.79 ± 1.44, *p* = 0.008) (Figure 1). Both A’ and S’ decreased in Groups I and II post-closure. The E/E’ was more elevated in Group II than in Group I at both pre-closure and post-closure (pre-closure: 9.60 ± 5.15 vs. 7.41 ± 1.42, *p* < 0.003; post-closure: 13.03 + 4.05 vs. 10.49 ± 3.95, *p* < 0.02) (Table 2), also E/E’ was significantly elevated at post-closure in Group I (pre-closure: 7.41 ± 1.42 vs. post-closure: 10.49 ± 3.95, *p* = 0.005) and II (pre-closure: 9.60 ± 5.15 vs. post-closure: 13.03 + 4.05, *p* = 0.005) (Figure 1). 

## 6. Discussion

Left-to-right inter-atrial shunting through the ASD causes right and left heart volume overload [5]. Even in asymptomatic patients, volume discrepancy and concomitant alteration between the left and right heart chambers progress with long-term shunting. 

In patients presenting with senile LV dysfunction, left-to-right shunt in ASD could offer another inter-atrial route for LA flow instead of LV filling, and mask the abnormal increase of LA pressure and LV filling pressure [7]. Therefore, in these patients, ASD closure can increase the LV diastolic and LV filling pressures in the enlarged, volume-loaded left heart. 

To exclude ASD patients with existing diastolic dysfunction, we included asymptomatic patients who believed that they did not have any heart diseases or cardiac dysfunction, and were incidentally diagnosed with ASD at routine health check-up without presenting with arrhythmias, right HF, or pulmonary hypertension. Surprisingly, the size of their ASDs were relatively large, and they presented with RV volume overload; the defect size was 21.77 + 6.79 mm and the balloon measurement was 24.29 ± 6.64 mm, certainly requiring treatment with device occlusion. 

ASD closure causes a rapid left ventricular (LV) volume overload and a consequent surge in myocardial oxygen consumption, because of the abrupt stoppage of the left-to-right shunt. These important hemodynamic changes are generally tolerated in patients with normal LV function [8], but may be detrimental in patients with LV dysfunction [9] and in elderly patients with decreased LV compliance [3].

As we compared the echocardiographic measurements taken before procedure and 2 years after ASD closure, the LVEDD and LV mass were observed to have increased, while the RV pressure had lowered after closure. With ASD closure, the LV changed from being a long-standing underloaded chamber to a relatively enlarged chamber that was as normal as could be expected, while the RV changed from being a volume-overloaded ventricle with an elevated pressure to a reasonably-sized chamber with a reduced volume. Meanwhile, LA volume and the LA volume index showed the increased measurement after closure, even it was without statistical significance, however, the older age patients showed the variation of re-decreasing LA volume and LA volume index (Figure 1), which might be due to age-related alteration of LA pressure with diastolic functional change.

Furthermore, the post-closure increase of E/E’ implied a diastolic function decline with relaxation abnormality in all the patients in younger age and older age group (Table 2). Diastolic dysfunction may progress after ASD closure; as opposed to in younger patients of Group I, ASD closure in older patients of Group II resulted in further deterioration of baseline LV relaxation and increased the age-related LV stiffness, thereby further increasing the LV filling pressure. Luckily, no patients developed severe congestive HF in this study. This could be an adaptive phenomenon of post-closure myocardial adjustment with increased volume loading.

Previous studies that examined changes in relaxation using echocardiographic parameters measured before and after ASD device repair yielded inconsistent results. Hanseus KC et al. reported no significant changes in relaxation 1 or 2 days after ASD closure in both, the pediatric [10] and wider populations [11]; conversely, other studies have reported a decrease in the tissue Doppler spectral values in both these populations [12,13]. Meanwhile, Makino K et al. reported that LV diastolic function in post-closure ASD patients during the chronic phase was the same as that in healthy subjects aged below 50 years [14], however, LA dimension was larger, and the tissue Doppler E’ and E/E’ were significantly increased in the post ASD closure in elderly group of more than 50 years old. We also evaluated the patients grouping with below and equal or above the age of 50, followed for 2 years, the results revealed significant findings between two groups.

As Tomai F et al. reported for the case of ASD related with LV dysfunction, a real-time preprocedural evaluation of the hemodynamic effects with left-to-right shunt elimination may be useful for decision-making [15]. Even though our patients from health check-up were all asymptomatic, if we considered whether an intra-procedural balloon stress-test may be useful in unmasking latent diastolic impairment during the procedure, it might be helpful for expecting the hemodynamic changes with procedure [16].

The most prominent and consistent hemodynamic change after transcatheter ASD closure, and subsequent elimination of the inter-atrial shunt flow with device occlusion, is an increase in the LV preload with a simultaneous decrease in the RV volume. If LV diastolic dysfunction already existed before ASD closure, the LV cannot handle this acute elevation in preload after closure, resulting in a marked escalation of the LV end-diastolic pressure and pulmonary congestion [17]. Furthermore, ventricular diastolic myocardial stiffness is another important factor that can impact ventricular diastolic performance. Despite challenges in quantifying the diastolic chamber stiffness using echocardiography, Chen CH et al. published a study in which invasive ventricular pressure-volume measurement revealed a significant increase in age-related diastolic myocardial stiffness [18].

Therefore, older patients experience prominent diastolic dysfunction after ASD closure more frequently than younger patients, as demonstrated by our study results. Moreover, even asymptomatic patients exhibited a similar diastolic function change after ASD closure, without presenting with any cardiac function deterioration patterns before diagnosis. We believe that ASD closure in elderly patients with suspected or existing LV or RV dysfunction should be performed only after a meticulous consideration of the hemodynamic benefits and risks of closure [7,19]. 

The limitation of this study, due to the retrospective review of the patients from the regular health check-up, did not include blood test, especially brain natriuretic peptide (BNP) data, quality of life data with cardiac function including diastolic assessment long-term prior to ASD closure. Although it is not possible to investigate the functional change and myocardial analysis, all the included patients were asymptomatic without any cardiac disease, such as arrhythmias, right HF, or pulmonary hypertension. Further study will be necessary with the prospective design for the various aspects, not only for the diastolic function, but also with the quality of life with functional class, and other various comorbidities including diabetes, liver or kidney issues, comparing with healthy controls.

## 7. Conclusions

The LA size and LV function exhibited recognizable remodeling after transcatheter ASD closure. Because the LV preload elevation (i.e., E/E’) after ASD closure can be pronounced in older patients, caution should be provided more in older patients than in younger patients. It may be beneficial to close the ASD at an earlier age in adults even after a late diagnosis; a relatively younger age may be more suited for adaptation to the remodeling process to protect the myocardial function. In addition, vigilant early care would be helpful for the previous underloaded left heart by considering for latent diastolic impairment with the procedure.

## Figures and Tables

**Table 1:** Comparison of echocardiographic data collected before and after atrial septal defect closure (N = 38).

N = 38	Pre-Closure	Post-Closure (2-Year Follow-Up)	*p*-Value
**LVEDD (mm)**	40.76 ± 3.28	43.39 ± 3.52	0.001
**LVESD (mm)**	26.86 ± 2.98	27.79 ± 3.11	0.11
**LVEF (%)**	66.41 ± 5.56	68.69 ± 5.08	0.48
**LV mass (g)**	99.64 ± 28.81	116.57 ± 32.03	0.01
**LV mass Ix (g/m^2^)**	61.23 ± 16.22	71.09 ± 16.07	0.01
**LA volume (mL)**	42.83 ± 15.67	45.48 ± 14.57	0.24
**LA vol Ix (mL/m^2^)**	26.1 ± 9.03	27.98 ± 8.99	0.20
**RVSP (mmHg)**	36.88 ± 12.20	31.81 ± 11.11	0.04
**Mitral E (cm/s)**	81.55 ± 19.54	84.79 ± 23.62	0.28
**Mitral A (cm/s)**	55.59 ± 12.90	60.68 ± 21.95	0.14
**DT (ms)**	191.68 ± 47.40	197.85 ± 50.62	0.31
**TDI E’ (cm/s)**	9.91 ± 2.87	8.12 ± 2.91	0.008
**TDI A’ (cm/s)**	8.83 ± 2.34	7.81 ± 1.72	0.03
**TDI S’ (cm/s)**	9.96 ± 2.55	8.06 ± 1.69	0.004
**E/E’**	8.79 ± 3.19	11.58 ± 4.80	0.005

LV: left ventricle, LA: left atrium, LVEDD: left ventricular end-diastolic dimension, LVESD: left ventricular end-systolic dimension, LVEF: left ventricular ejection fraction, RV: right ventricle, RVSP: right ventricular systolic pressure, DT; Deceleration time, E; peak early inflow velocity, A; peak late inflow velocity, TDI: tissue Doppler imaging, E’; peak early diastolic velocity, A’; peak late diastolic velocity, S’: peak systolic velocity.

**Table 2:** Comparison of echocardiographic data collected before and after atrial septal defect closure in Group I (N = 18) and Group II (N = 20).

	Group I (<50 Years Old) (*n* = 18)	Group II (≥50 Years Old) (*n* = 20)	*p*-Value
**Age (years)** **LVEDD pre**	33.06 ± 9.43 40.44 ± 3.50	62.55 ± 7.54 41.15 ± 3.08	NA 0.28
**post**	44.43 ± 3.44	42.63 ± 3.47	0.07
**LVESD pre**	26.69 ± 2.87	27.08 ± 3.23	0.36
**post**	28.5 ± 3.41	27.26 ± 2.84	0.13
**LVEF pre**	66.19 ± 5.94	66.69 ± 5.28	0.40
**post**	68.86 ± 6.01	68.58 ± 4.44	0.43
**LV mass pre**	89.69 ± 21.43	111.89 ± 32.68	0.02
**post**	113.24 ± 35.5	119.02 ± 29.97	0.31
**LV mass Ix pre**	54.93 ± 11.48	68.98 ± 18.2	0.008
**post**	68.73 ± 21.18	72.83 ± 12.75	0.24
**LA volume pre**	36.07 ± 10.24	51.15 ± 17.49	0.003
**post**	38.16 ± 15.87	50.87 ± 11.09	0.005
**LA volume Ix pre**	21.89 ± 6.31	31.28 ± 9.37	0.001
**post**	23.17 ± 9.88	31.52 ± 6.48	0.003
**RVSP pre**	33.14 ± 6.97	41.47 ± 15.66	0.03
**post**	25.18 ± 6.31	36.6 ± 11.48	0.001
**Mitral E pre**	84.06 ± 17.2	82.71 ± 22.5	0.17
**post**	78.46 ± 22.37	86.32 ± 24.88	0.33
**Mitral A pre**	50.56 ± 11.17	49.78 ± 23.84	0.005
**post**	62.9 ± 12.08	69.64 ± 15.88	0.004
**DT pre**	189.67 ± 48.19	208.71 ± 65.9	0.40
**post**	194.00 ± 48.33	189.84 ± 35.5	0.15
**TDI E’ pre**	11.53 ± 2.38	9.92 ± 3.45	0.0001
**post**	7.93 ± 2.09	6.79 ± 1.44	0.0005
**TDI A’ pre**	8.09 ± 2.36	7.14 ± 1.29	0.02
**post**	9.91 ± 1.92	8.35 ± 1.87	0.02
**TDI S’ pre**	11.09 ± 2.55	8.86 ± 1.95	0.002
**post**	8.57 ± 1.80	7.47 ± 1.22	0.008
**E/E’ pre**	7.41 ± 1.42	9.60 ± 5.15	0.003
**post**	10.49 ± 3.95	13.03 ± 4.05	0.02

LV: left ventricle, LA: left atrium, LVEDD: left ventricular end-diastolic dimension, LVESD: left ventricular end-systolic dimension, LVEF: left ventricular ejection fraction, RV: right ventricle, RVSP: right ventricular systolic pressure, DT; Deceleration time, E; peak early inflow velocity, A; peak late inflow velocity, TDI: tissue Doppler imaging, E’; peak early diastolic velocity, A’; peak late diastolic velocity, S’: peak systolic velocity.
